# Dosimetric comparison between cone/Iris‐based and InCise MLC‐based CyberKnife plans for single and multiple brain metastases

**DOI:** 10.1120/jacmp.v17i5.6260

**Published:** 2016-09-08

**Authors:** Si Young Jang, Ron Lalonde, Cihat Ozhasoglu, Steven Burton, Dwight Heron, M. Saiful Huq

**Affiliations:** ^1^ Department of Radiation Oncology The University of Pittsburgh Cancer Institute Pittsburgh PA USA

**Keywords:** CyberKnife, SRS, InCise MLC, dosimetric comparison

## Abstract

We performed an evaluation of the CyberKnife InCise MLC by comparing plan qualities for single and multiple brain lesions generated using the first version of InCise MLC, fixed cone, and Iris collimators. We also investigated differences in delivery efficiency among the three collimators. Twenty‐four patients with single or multiple brain mets treated previously in our clinic on a CyberKnife M6 using cone/Iris collimators were selected for this study. Treatment plans were generated for all lesions using the InCise MLC. Number of monitor units, delivery time, target coverage, conformity index, and dose falloff were compared between MLC‐ and clinical cone/Iris‐based plans. Statistical analysis was performed using the nonparametric Wilcoxon‐Mann‐Whitney signed‐rank test. The planning accuracy of the MLC‐based plans was validated using chamber and film measurements. The InCise MLC‐based plans achieved mean dose and target coverage comparable to the cone/Iris‐based plans. Although the conformity indices of the MLC‐based plans were slightly higher than those of the cone/Iris‐based plans, beam delivery time for the MLC‐based plans was shorter by 30%∼40%. For smaller targets or cases with OARs located close to or abutting target volumes, MLC‐based plans provided inferior dose conformity compared to cone/Iris‐based plans. The QA results of MLC‐based plans were within 5% absolute dose difference with over 90% gamma passing rate using 2%/2 mm gamma criteria. The first version of InCise MLC could be a useful delivery modality, especially for clinical situations for which delivery time is a limiting factor or for multitarget cases.

PACS number(s): 87.53.Ly, 87.55.D‐

## I. INTRODUCTION

Stereotactic radiosurgery (SRS) or fractionated stereotactic radiotherapy (SRT) are core treatment modalities for patients with brain metastases or benign tumors.^1^, ^2^, ^3^ SRS treatment is a complex procedure involving frame‐based or frameless immobilization and multiple small beams delivered in noncoplanar fashion. Radiation delivery of small beams using multiple noncoplanar arcs or beams projected on the target regions results in a highly conformal dose distribution around the targets while minimizing dose to the normal brain tissue.^4^, ^5^ Submillimeter accuracy can be achieved, and a maximum error of 1 mm is commonly considered as an acceptable accuracy in terms of the target localization.^6^, ^7^

In addition to delivery accuracy for SRS or SRT, it is important to consider the delivery efficiency (i.e., treatment time in a single session) during the planning stage, since some patients cannot be laid on the treatment couch for a long period of time due to pain.^8^, ^9^, ^10^ Those patients who have difficulty staying on the treatment couch are ideal candidates for treatment using a multi‐leaf collimator (MLC) or volumetric‐modulated arc therapy (VMAT) technique that is capable of delivering treatment in a short period of time.^8^, ^11^

In our clinic, we treat intracranial lesions using either fixed‐cones or Iris collimators mounted on the CyberKnife M6 (Accuray Inc., Sunnyvale, CA), which employs a pair of orthogonal kV X‐ray imagers to localize targets during beam delivery. Typical delivery times range from 30 to 90 min, depending on the number of targets, the type of collimators, and the complexity of target shapes. With the emergence of technologies such as Leksell Gamma Knife Perfexion (Elekta AB, Stockholm, Sweden), CyberKnife or linear‐accelerator‐based modalities, it is becoming a standard practice in many clinics to generate a single SRS plan for multiple brain lesions and deliver treatment in a single session.^(12)^ Since its installation, we have been using the CyberKnife M6 on a daily basis to deliver multifocal treatments.

Recently, Accuray released a new collimator, the InCise MLC, for clinical use with the CyberKnife M6. The first version of InCise (InCise1) MLC consists of 41 leaves projecting a width of 2.5 mm at a plane located at 800 mm source‐to‐axis distance (SAD). Flattening filter‐free photon beam of 6 MV energy can be delivered with the nominal dose rate of 1,000 monitor units (MU)/min. Since the maximum size of MLC fields projected at 800 mm SAD is 110mm±97.5mm, there is some advantage in using MLC for large and irregularly shaped targets with regard to delivery time.^(13)^ However, the minimum MLC opening is limited to 7.6mm±7.5mm.^(14)^ An additional advantage of the InCise1 MLC compared to existing MLC models, such as HD120 MLC (Varian Inc., Palo Alto, CA), is that the transmission through the InCise1 MLC leaves is less than 0.5%.^(14)^ This is accomplished by utilizing tungsten leaves of thickness 90 mm. Furthermore, the specification of leaf positioning accuracy is below 0.5 mm^(14)^ at 800 mm SAD, resulting in submillimeter accuracy of beam delivery.

In this study, we compared the dosimetric and delivery parameters of the InCise1 MLC‐based plans for intracranial lesions with those of the cone/Iris‐based treatment plans that had been used clinically for patient treatments. We also evaluated the capability of MLC for treating multiple targets, large and/or irregularly shaped targets (i.e., postsurgical or acoustic cases), with or without an organ at risk (OAR) next to the lesions.

## II. MATERIALS AND METHODS

### A. Patient characteristics

Twenty‐four patients with single or multiple intracranial lesions were selected for this retrospective study. All patients were treated in our clinic using either the fixed cones or Iris collimator on the M6 system. We selected patients randomly who had been treated prior to the establishment of the InCise1 MLC to minimize any bias in terms of planning and delivery aspects. Tumor characteristics for all patients are summarized in Table 1. Single and multiple lesions of both small and large volumes were chosen deliberately to evaluate the capabilities of the InCise1 MLC in terms of plan quality and delivery efficiency. Postsurgical and acoustic cases with irregularly shaped targets were also selected. The median volume of all lesions in this study was $2.5\;{\text{cm}}^{3}$, with a range from $0.09\;{\text{cm}}^{3}$ to $47.0\;{\text{cm}}^{3}$. Of the 24 patients selected, 16 patients had a single lesion with volumes ranging from $0.3\;{\text{cm}}^{3}$ to $47.0\;{\text{cm}}^{3}$, and 8 patients had multiple lesions of 2 to 8 targets with total volumes ranging from $0.09\;{\text{cm}}^{3}$ to $29.4\;{\text{cm}}^{3}$. One patient had eight lesions with two plans: one plan included two targets and the other included six targets. Seven cases had OARs such as brainstem or optic chiasm abutting with or close to the lesions. One case had a lesion inside the brainstem. The prescription isodose line (IDL) for all cases was 80%. The prescription dose varied from 15 Gy to 25 Gy with 1 to 5 fractions.

**Table 1:** Summary of patient characteristics. Target volume data represent median (minimum volume ~ maximum volume).

Number of patients	24 patients (25 plans)
‐ single target	‐ 16 patients
‐ 2 ~ 4 targets	‐ 6 patients
‐ >4 targets	‐ 2 patients
‐ circular shape	‐ 18 plans
‐ irregular shape of target or abutting with OARs	‐ 7 plans
‐ $\text{target volume}\leq 1{\text{cm}}^{3}$s	‐ 7 plans
‐ $1{\text{cm}}^{3}<\text{target volume}\leq 5{\text{cm}}^{3}$	‐ 9 plans
‐ $\text{target volume}>5{\text{cm}}^{3}$	‐ 9 plans
Target volumes (cm^3^)	2.5(0.09∼47.0)
‐ single target	−2.3(0.3∼47.0)
‐ multiple targets	−2.7(0.09∼29.4)
‐ circular shape	−2.2(0.09∼11.1)
‐ irregular shape of target or abutting with OARs	−11.1(0.3∼47.0)
‐ $\text{target volume}\leq 1{\text{cm}}^{3}$	−0.3(0.09∼0.6)
‐ $1{\text{cm}}^{3}<\text{target volume}\leq 5{\text{cm}}^{3}$	−2.3(1.2∼4.4)
‐ $\text{target volume}>5{\text{cm}}^{3}$	−14.7(9.1∼47.0)

OAR=organ at risk

### B. MLC‐based SRS treatment planning

**Figure 1:**
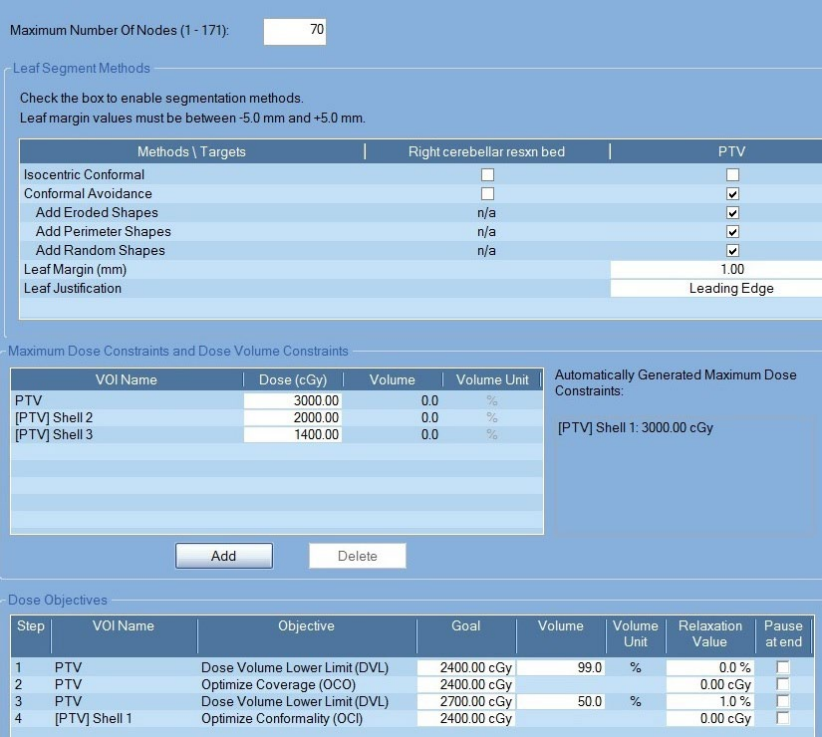
An example of sequential optimization script used for an irregularly shaped target. The size of target was $18.3\;{\text{cm}}^{3}$ and the prescription dose was 24 Gy in 3 fractions. Three different sizes of shells located at 2 mm, 5 mm, and 10 mm away from the target boundary were used for MLC‐based and cone/Iris‐based plans.

All treatment plans were generated using Multiplan treatment planning software version 5.1.3 (Accuray Inc.). Computed tomography (CT) images of 1.25 mm slice thickness were used as primary planning CT images. Magnetic resonance imaging (MRI) images of 1.2 mm slice thickness were fused with the CT images for delineating target volumes. All 24 patients treated with fixed‐cone or Iris‐based clinical plans were replanned with the InCise1 MLC. For MLC shaping, conformal avoidance targeting was selected, and all shapes of segments such as perimeter segment, eroded segment, and random segment were applied for the optimization. Depending on the complexity of the target, the maximum margin between the target volume and leading edge of the MLC leaves was varied between −1 to 1 mm. The sequential optimization algorithm^(15)^ was used for beam optimization with the Finite‐Sized Pencil‐Beam (FSPB) model for dose calculation. Figure 1 shows a sequential optimization script used for an irregularly shaped target. Tissue heterogeneity correction was used for all plans.

The total number of segments generated by the treatment planning system depends on the number of nodes selected, the number of avoidance regions that intersect the target, and the types of segment shapes chosen. We set the desired maximum number of nodes between 50 and 170. For multiple targets, we increased the number of nodes over 100 to get a better target coverage. Likewise, we reduced the number of nodes for cases with small‐size single targets. However, the actual number of beams was determined by the optimization algorithm itself and was slightly different from the desired values set by the planner. The number of segments per beam in the solution varied between 1 and 2. The maximum MU per segment was set between 150 and 350, and the maximum MU per node was approximately 1.5 times the maximum MU per segment.

Sensitivity analysis was performed by varying number of nodes, MU per segment, and MU per node to find out the impact of the number of nodes and segments on the plan quality and treatment time. In this study, the MU per segment with the same number of nodes was varied from 50 MU/segment up to 150 MU/segment, and the number of nodes with the same MU/segment was varied from 30 to 70 to mimic intensity‐modulated radiation therapy (IMRT) in step‐and‐shoot mode.

For dosimetric comparison, the prescription dose and dose constraints to OARs that had been used for clinical fixed cone/Iris‐based plans were utilized for the MLC‐based plans. Furthermore, the same tuning structures or rings were used to improve dose conformity and dose falloff outside the target volumes. This way of optimization minimizes any subjective issues on plan comparison.

Plan quality was evaluated by comparing dosimetric indices such as minimum and mean target dose ($D_{\text{min}},D_{\text{mean}}$) and target coverage. The maximum target dose was not included for comparison since in our clinic we set the prescription dose to the 80% IDL of the maximum target dose; therefore, the maximum target dose is identical between MLC‐ and cone/Iris‐based plans. The level of dose conformity was evaluated using the dose conformity index (CI) of the Radiation Therapy Oncology Group (RTOG), which is defined as the ratio of the volume receiving the prescription dose or greater and the volume of the target.^(16)^ For the OARs such as the brainstem and optic chiasm, $D_{\text{mean}}$ and $D_{\text{max}}$ were compared.

The capability of MLC leaves to produce a sharp dose gradient was evaluated by computing the RTOG quality matrix for dose gradient ($R_{50\%}$) which is represented by the ratio of the volume covered by the 50% IDL of the maximum target dose ($D_{50\%}$) to the target volume.^17^, ^18^ $R_{50\%}$ was computed using the 50% IDL of the maximum target dose rather than 50% prescription dose for plan comparison purpose. Dose falloff to the low‐dose region ($R_{10\%}$), which is represented by the ratio of the volume covered by the 10% IDL of the maximum target dose ($D_{10\%}$) to the target volume, was also computed. $R_{10\%}$ is a measure of the impact of MLC leakage as well as of radiation penumbra, which is a calculated quantity rather than a measurable quantity. The number of nodes and segments for the MLC‐based plans were compared with the nodes for the cone/Iris‐based plans. Finally, total MU and delivery time between MLC‐ and cone/Iris‐based plans were compared to find out the clinical benefits of the MLC‐based plans in terms of treatment delivery efficiency.

Statistical analysis with the nonparametric Wilcoxon‐Mann‐Whitney signed‐rank test was performed to compare the dosimetric indices between MLC‐ and cone/Iris‐based plans. The R Project for Statistical Computing (The R Foundation) was used, and the threshold for statistical significance was set to p≤0.05.^(19)^

### C. Phantom quality assurance (QA) for MLC‐based plans

The accuracy of InCise1 MLC‐based planning and delivery was evaluated by delivering 10 MLC‐based treatment plans to a stereotactic dose verification QA phantom (Standard Imaging, Middleton, WI). A small‐volume ionization chamber (PTW pinpoint chamber, $\text{volume}=0.015{\text{cm}}^{3}$; PTW, Freiburg, Germany) and Gafchromic EBT3 film (ISP Inc., Wayne, NJ) were inserted into the phantom to measure the central dose of the target and relative dose distribution, respectively. The active volume of the ionization chamber was positioned in low‐dose gradient regions within target volumes, but the location of the lesion in QA plans relative to the imaging center was identical to that of the clinical plans.

The delivered dose was scaled down from the prescription dose such that a dose in the range of 100 cGy to 600 cGy was delivered to the film plane. The film was registered to the treatment plan,^(20)^ and postirradiation waiting time was 24 hrs. The EBT3 films were scanned using the Epson Expression 11000XL flatbed scanner (US Epson, Long Beach, CA) and analyzed using FilmQA Pro 2014 software (Ashland Inc., Covington, KY). Acceptance criteria for dosimetric accuracy were ≤ 5% for the point‐dose measurement and ≥90% gamma passing rate using 2%/2 mm for the gamma evaluation of local dose difference and distance‐to‐agreement, respectively.^21^, ^22^ In our clinic, we compute the gamma with a threshold of 25% IDL due to uncertainty in the low‐dose region for film dosimetry. In this study, however, we evaluated the dose distribution in the low‐dose region without setting any IDL threshold with a H&D curve for EBT3 film measured from 1,000 cGy down to 30 cGy.

In addition to the phantom QA of MLC‐based plans, we performed an irradiation of the Imaging and Radiation Oncology Core (IROC) Houston's Head Neck IMRT Phantom as part of a credentialing process, and the phantom irradiation satisfied the criteria established by the IROC Houston in collaboration with the cooperative study groups.

## III. RESULTS

### A. Summary of dosimetric indices

**Figure 2:**
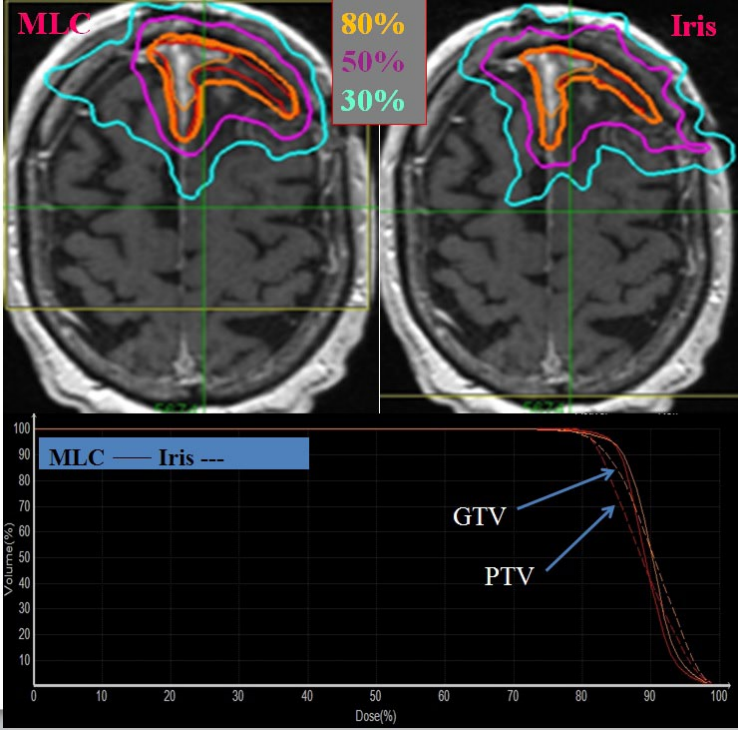
Axial dose distribution (top) and dose‐volume histogram (bottom) of MLC‐based and cone/Iris‐based CyberKnife plan. The target lesion is shown in red color and isodose lines are shown as contours. Solid and dotted lines on the dose‐volume histogram represent MLC‐based and cone/Iris‐based plan, respectively. It was postsurgical case with an irregularly shaped target, and the prescription dose was 25 Gy in 5 fractions.

Figure 2 compares IDL and dose‐volume histogram (DVH) for MLC‐based and cone/Iris‐based plans for a selected patient. Table 2 summarizes dosimetric indices for all 24 patients. The median ratios for $D_{\text{min}},D_{\text{mean}}$, and target coverage between the MLC‐based and cone/Iris‐based plans were 0.91, 1.01, and 1.00, respectively. A ratio of dosimetric index greater than 1 indicates that the MLC‐based plan yields a higher value for that dosimetric index than does the cone/Iris‐based plan. The variations in $D_{\text{min}},D_{\text{mean}}$, and target coverage between the MLC‐based and cone/Iris‐based plans were within 1 SD of each other, and the MLC delivery modality achieved comparable dosimetric parameters to those of cone/Iris‐based modality. Since the prescription line to the target was always set to 80% IDL of the maximum target dose, the homogeneity index (HI) was 1.25 regardless of the planning techniques. For cases in which the OARs were abutted with a target and/or the dose constraints were challenging to meet, the cone/Iris‐based plans were better than the MLC‐based plans in terms of target coverage with comparable dose to OARs.

Table 2 also shows the summary of dose conformity between the MLC‐based plans and cone/Iris‐based plans. A ratio of greater than 1 indicates that the cone/Iris‐based plan shows better dose conformity than does the MLC‐based plan. For all plans, the median CI was 1.37 for MLC‐based plans and 1.28 for cone/Iris‐based plans. CI values of the MLC‐based plans were higher than those of the cone/Iris‐based plans regardless of volume of target, number of targets, and complexity of the target shape. Statistically, the difference in CI between MLC‐based and cone/Iris‐based plans was significant for all cases; however, this was not the case when data were stratified according to single versus multiple lesions, volume of target, and irregularity of target.

For cases with complex target shape or target abutting with the OARs, the median ratio of CI value between the MLC‐based plans and cone/Iris‐based plans was 1.13, while the median ratio of target coverage was 0.99. This suggests that MLC‐based plans might be less favorable for cases where conformal dose distribution is a limiting factor. Overall, the MLC‐based plans provided less conformity than the cone/Iris‐based plans. With MLC‐based plans for irregularly shaped targets or abutting with the OARs, the CI values of the MLC‐based plans were higher than those of the cone/Iris‐based plans.

**Table 2:** Summary of dosimetric results for targets in CyberKnife InCise MLC and cone/Iris plans.

	*Number of Plans*	*Median Target Volume (cc)*	*CI*			*Ratio (MLC/cone or Iris)*			
			*Cone/Iris*	*MLC*	*Wilcoxon signed‐rank p*	$D_{\text{min}}$	$D_{\text{mean}}$	*Coverage*	*CI*
All cases	25	2.5	1.34±0.28	1.44±0.30	0.035	0.95±0.06	1.01±0.01	0.99±0.02	1.08±0.10
			(1.28)^a^	(1.37)^a^		(0.91)^a^	(1.01)^a^	(1.00)^a^	(1.05)^a^
single target	16	2.3	1.26±0.14	1.34±0.16	0.105	0.94±0.05	1.00±0.01	0.99±0.02	1.07±0.09
			(1.24)	(1.30)		(0.93)	(1.00)	(1.00)	(1.03)
multiple targets	9	2.7	1.49±0.40	1.61±0.40	0.057	0.95±0.07	1.01±0.01	0.99±0.02	1.09±0.06
			(1.33)	(1.50)		(0.96)	(1.01)	(1.00)	(1.07)
circular shape	18	2.2	1.37±0.32	1.44±0.34	0.125	0.96±0.04	1.01±0.01	0.99±0.02	1.05±0.06
			(1.29)	(1.37)		(0.97)	(1.01)	(1.00)	(1.04)
irregular shape of target or abutting with OARs	7	11.1	1.27±0.16	1.43±0.19	0.097	0.91±0.08	1.00±0.01	0.99±0.01	1.13±0.10
			(1.22)	(1.45)		(0.92)	(1.00)	(0.99)	(1.13)
target volume ≤1cc	7	0.3	1.36±0.15	1.42±0.14	0.522	0.98±0.04	1.00±0.02	1.00±0.02	1.05±0.09
			(1.33)	(1.36)		(1.00)	(1.00)	(1.00)	(1.05)
1cc<target volume≤5cc target volume>5cc	9	2.3	1.30±0.09	1.38±0.11	0.052	0.95±0.06	1.01±0.01	0.98±0.02	1.07±0.06
			(1.28)	(1.39)	(0.95)	(1.01)	(0.99)	(1.07)
target volume>5cc	9	14.7	1.38±0.46	1.51±0.48	0.185
		0.93±0.07	1.01±0.01	1.00±0.01	1.10±0.10
(1.18)	(1.29)	(0.93)	(1.00)	(1.00)	(1.06)

^a^ Data represent mean ± SD (median).

CI=conformity index; coverage=target dose coverage; $D_{\text{min}},D_{\text{mean}}$ = minimum and mean dose to target; OAR=organ at risk.

### B. Dose falloff with varying distance from target

Table 3 summarizes the dose falloff for the MLC and cone/Iris‐based plans, which is characterized with the $R_{50\%}$ and $R_{10\%}$. Overall, high dose gradient was achieved between the target periphery and the 50% IDL for both the MLC‐based and cone/Iris‐based plans. Median $R_{50\%}$ values for the MLC‐ and cone/Iris‐based plans for all cases were 3.4 and 3.7, respectively. Except for irregularly shaped targets or abutting with the OARs, the ratio of $R_{50\%}$ between MLC‐based plans and cone/Iris‐based plans was less than 1 regardless of the volume of target and number of target lesions; however, this difference was not statistically significant. A ratio of less than 1 indicates that the MLC‐based plan shows better dose falloff than does the cone/Iris‐based plan.

For irregularly shaped targets or abutting with the OARs, the ratio of $R_{50\%}$ was slightly higher than 1. For small targets, $R_{50\%}$ for cone/Iris‐based plans was slightly better than that for MLC‐based plans. For all cases, the median values of $R_{10\%}$ for the MLC‐based and cone/Iris‐based plans were 38.8 and 42.7, respectively, which shows that the distance to 10% IDL decreases as the number of beams is reduced by using the InCise1 MLC. However, the difference in $R_{10\%}$ was statistically not significant. For multiple targets with greater than two lesions, $R_{10\%}$ was comparable to that of single targets.

**Table 3:** Summary of dosimetric results in terms of dose falloff in CyberKnife InCise MLC‐based and cone/Iris‐based plans.

	*Number of Plans*	$R_{50\%}$		$R_{10\%}$		*Wilcoxon signed‐rank p*		*Ratio (MLC/cone or Iris)*	
		*Cone/Iris*	*MLC*	*Cone/Iris*	*MLC*	$R_{50\%}$	$R_{10\%}$	$R_{50\%}$	$R_{10\%}$
All cases	25	3.8±1.1	3.6±1.0	48.7±25.7	48.3±30.3	0.5 35	0.7 58	0.94±0.13	0.98±0.21
		(3.7)^a^	(3.4)^a^	(42.7)^a^	(38.8)^a^			(0.94)^a^	(0.96)^a^
single target	16	3.6±1.0	3.3±0.9	37.5±13.4	36.2±14.0	0.435	0.804	0.93±0.15	0.97±0.20
		(3.4)	(3.2)	(37.6)	(34.9)			(0.90)	(0.89)
multiple targets	9	4.2±1.1	4.1±1.0	65.1±31.6	67.2±38.7	0.8 98	0.8 47	0.97±0.12	1.02±0.21
		(4.2)	(4.7)	(73.4)	(64.0)			(0.96)	(0.99)
circular shape	18	3.9±1.2	3.5±1.1	51.0±25.2	47.3±26.50	0.3 51	0.5 84	0.90±0.12	0.92±0.18
		(3.7)	(3.2)	(46.6)	(40.5)			(0.90)	(0.89)
irregular shape of target or abutting with OARs	7	3.6±0.6	3.8±0.7	42.5±27.7	50.8±41.00	0.805	0.805	1.05±0.11	1.12±0.21
		(3.7)	(3.8)	(30.0)	(33.2)			(1.06)	(1.17)
target volume ≤1cc	7	4.7±0.9	4.5±0.7	62.2±14.3	60.8±21.2	0.902	0.805	0.97±0.11	0.97±0.23
		(4.6)	(4.8)	(63.4)	(51.7)			(0.97)	(0.81)
1cc<target volume≤5cc	9	3.7±1.1	3.5±0.9	59.2±31.5	61.9±37.9	1.000	0.796	0.97±0.14	1.04±0.25
		(3.5)	(3.2)	(45.7)	(51.8)			(0.95)	(0.99)
target volume>5cc	9	3.2±0.6	2.9±0.9	27.6±6.70	25.0±5.7 (24.2)	0.1 85	0.4 89	0.90±0.15	0.92±0.14
		(3.0)	(2.5)	(27.5)	(42.2)			(0.87)	(0.90)

^a^ Data represent mean ± SD (median).

OAR=organ at risk.

### C. Dose to critical organ

**Figure 3:**
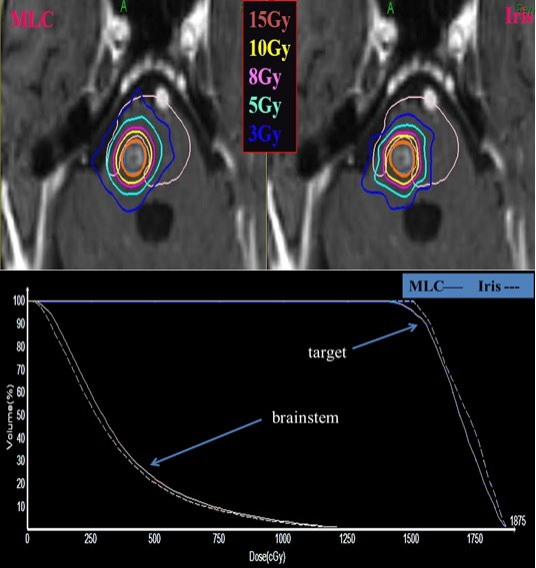
Axial dose distributions (top) and dose‐volume histogram (bottom) of MLC‐based and cone/Iris‐based CyberKnife plan. The target lesion is shown in red color and isodose lines are shown as contours. Solid and dotted lines on the dose‐volume histogram represent MLC‐based and cone/Iris‐based plan, respectively. It was a case with a circular‐shaped target abutting with brainstem, and the prescription dose was 15 Gy in 1 fraction.

The average ratios of mean and maximum doses to the OAR between MLC‐based and cone/Iris‐based plans were 0.95±0.27 and 0.95±0.13, respectively. The median ratios of mean and maximum doses to the OAR between the MLC‐based and cone/Iris‐based plans were 0.96 and 1.00, respectively. A ratio greater than 1 indicates that the MLC‐based plan shows a higher dose to OAR than does the cone/Iris‐based plan. For the cases that dose constraint to OAR was a limiting factor rather than target coverage, the ratio of dose to the OAR between the MLC‐based and cone/Iris‐based plans was close to 1 since the dose constraint to the OAR was considered to be the primary factor to be met. Although the MLC‐based plans were comparable to the cone/Iris‐based plans in terms of dose sparing of OARs, the target coverages of the MLC‐based plans for targets abutting with OARs were inferior to those of the cone/Iris‐based plans, as shown in Fig. 3.

### D. Beam delivery efficiency

Table 4 shows the summary of beam delivery efficiency between MLC‐based and cone/Iris‐based plans. For all cases, the median MUs for the MLC‐based and cone/Iris‐based plans were 9,091 and 17,041, respectively. The median delivery time was 58 min for the cone/Iris‐based plans and 36 min for the MLC‐based plans. The delivery time estimated by the MultiPlan includes 5‐minute setup time, robot travelling time, beam‐on time, and imaging time with 1‐minute interval. The total MU for the MLC treatment plans was considerably reduced by >50% compared with the cone/Iris‐based plans. As shown in Table 4, this reduction in total MU resulted from the reduction in total number of beams in the MLC‐based plans (median number of nodes=58) compared to the cone/Iris‐based plans (median number of nodes=166) since each beam consists of multiple segments depending on the complexity of target shape. As the volume of target increased, the total MU and delivery time increased, while the ratio of delivery time between MLC and cone/Iris‐based plans showed small variations. Statistically, the differences in MU, number of beams, and delivery time between MLC and cone/Iris‐based plans were significant for all cases regardless of number of lesions, volume of target, and irregularity of target.

**Table 4:** Summary of dosimetric results in terms of beam delivery efficiency in CyberKnife InCise MLC‐based and cone/Iris‐based plans. Data represent mean±SD (median). The number of plans for each category are identical to Table 3. Wilcoxon signed‐rank *p* was below 0.05 for all cases.

	*MU*		*Delivery Time (min)*		*Number of Beams*			*Ratio (MLC/cone or Iris)*	
	*Cone/Iris*	*MLC*	*Cone/Iris*	*MLC*	*Cone/Iris*	*MLC beam*	*MLC Segment*	*MU*	*Delivery Time (min)*
All cases	21275±10985	8971±5489	58±18	37±12	182±85	63±34	105±64	0.42±0.16	0.65±0.12
	(17041)	(9091)	(58)	(36)	(166)	(58)	(86)	(0.40)	(0.66)
single target	18149±11091	6709±3496	51±14	32±9	168±94	50±25	88±63	0.40±0.18	0.65±0.14
	(15178)	(4752)	(51)	(31)	(151)	(36)	(52)	(0.36)	(0.68)
multiple targets	26832±8777	12993±6248	72±15	46±13	207±61	86±37	135±58	0.47±0.10	0.64±0.07
	(26441)	(11727)	(67)	(44)	(202)	(73)	(138)	(0.49)	(0.65)
circular shape	18355±8115	8325±4976	56±17	36±11	168±17	61±33	94±58	0.45±0.16	0.66±0.12
	(16806)	(6572)	(56)	(33)	(56)	(49)	(86)	(0.43)	(0.66)
irregular shape of target or abutting with OARs	28782±14309	10634±6772	65±19	40±17	218±112	68±38	132±76	0.37±0.15	0.61±0.11
	(26442)	(10902)	(62)	(40)	(217)	(65)	(145)	(0.30)	(0.65)
target volume≤1cc	14606±5617	6329±3651	47±15	31±8	108±49	43±17	53±27	0.42±0.10	0.68±0.13
	(13280)	(3973)	(46)	(29)	(110)	(37)	(47)	(0.40)	(0.66)
1cc<target volume≤5cc	22795±11122	9671±8267	64±21	38±18	177±65	66±49	95±67	0.37±0.14	0.58±0.11
	(16666)	(4845)	(62)	(32)	(156)	(35)	(53)	(0.30)	(0.61)
target volume>5cc	24941±12569	10326±4819	61±13	41±6	245±79	74±20	156±45	0.49±0.20	0.69±0.11
	(21632)	(10902)	(62)	(40)	(242)	(65)	(145)	(0.52)	(0.69)

MU = monitor unit; OAR=organ at risk.

Although the ratio of MU (i.e., MU of MLC plan divided by MU of Iris or cone plan) was approximately 0.2∼0.7, the ratio of beam delivery time was 0.5∼0.9, showing that robot movement makes a substantial contribution to overall treatment rather than beam‐on time. Beam delivery time depended on total number of beams rather than total MU. MLC‐based plans had about 35% fewer beams than cone/Iris‐based plans, and beam delivery time for the MLC‐based plans was subsequently reduced by 30%∼40% compared with the cone/Iris‐based plans.

### E. Sensitivity analysis

**Figure 4:**
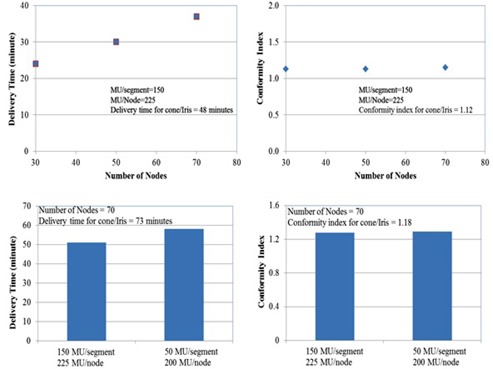
Results of sensitivity analysis with varying number of nodes, MU/segment, and MU/node. Top figures show that the delivery time of the MLC‐based plan increases linearly with varying number of nodes from 30 to 70. However, the conformity index is insensitive to the varying number of nodes. Bottom figures show an increasing delivery time in step‐and‐shoot style MLC delivery with a small MU/segment of 50 and a large MU/node of 200. However, the variation of conformity index is minimal.

Figure 4 shows the results of sensitivity analysis with varying number of nodes, MU/segment, and MU/node. As the number of desired nodes in optimization increased from 30 to 70, beam delivery time consequently increased without any improvement in dose conformity. By mimicking IMRT delivery using a small MU/segment of 50 MU/segment and a large MU/node of 200 MU/node, the beam delivery time of the MLC‐based plan was increased comparably to that of the cone/Iris‐based plan. However, there was a minimal CI variation when step‐and‐shoot style IMRT delivery was simulated.

### F. Dosimetric validation of MLC‐based plans

Table 5 shows the summary of dosimetric validation for 10 MLC‐based plans. The calculated doses in QA plans were in good agreement with the measured doses with ion chamber. The agreement between calculated dose to the target and measurements was in the range of −4.7% to 1.6%. The mean and median dose differences between the measured and calculated values for all 10 cases were −0.8% and −0.2%, respectively. The median and mean gamma pass rates for the 10 cases were 98.7% and 99.5%, respectively. The gamma passing rate for 10 cases was over 90% for all cases.

**Table 5:** Summary of dosimetric validation for 10 MLC‐based plans. Acceptance criteria for dosimetric accuracy were ≤5% for the point‐dose measurement and ≥90% gamma passing rate using 2%/2 mm for the gamma evaluation of local dose difference and distance to agreement, respectively.

*Case #*	*Number of MLC Segments*	*Estimated QA Delivery Time (min)*	*Ion Chamber Error (%)*	*Gamma (%)* (2%/2 mm)
1	12	18	−1.1	98.5
2	29	23	−4.7	99.9
3	29	29	−3.3	98.0
4	34	21	−0.8	96.5
5	48	34	0.4	100.0
6	81	25	1.6	94.8
7	131	38	1.4	99.9
8	138	44	0.9	99.6
9	170	45	−2.8	100.0
10	215	45	0.9	99.4
Avg	89	32	−0.8	98.7
SD	67	10	2.1	1.7
Med	65	32	−0.2	99.5

Avg=average,SD=standard deviation,Med=median.

## IV. DISCUSSION

Results of the present study showed that the InCise1 MLC‐based treatment planning was comparable to cone/Iris‐based treatment planning with respect to mean target dose and target coverage for intracranial tumors. The present results also showed that cone/Iris‐based plans yielded slightly lower CI values than the MLC‐based plans, indicating that the cone/Iris‐based plans are more conformal than the MLC‐based plans. For irregularly shaped targets, the observed differences in CI between the two modalities were not negligible, and these differences could not be reduced by increasing the number of beams in the MLC‐based plans. However, it should be noted that the CI values for the MLC‐based plans ranged from 1 to 2, which met the RTOG guideline.^(13)^ Since the minimum MLC opening is limited to 7.6mm±7.5mm ($>56.25\;{\text{mm}}^{2}$), very small targets (size <7.6mm×7.5mm) might not be candidates for MLC‐based planning.

In addition, LoSasso et al.^(23)^ reported that the error in delivered dose versus leaf position accuracy in the sliding window technique of MLC delivery was up to 5% for a gap width of 1 cm and gap error of 0.5 mm. LoSasso and colleagues also mentioned that the magnitude of dose error for the sliding window technique applied to the step‐and‐shoot technique of MLC delivery. Even with the specification of InCise1 MLC positioning accuracy of below 0.5 mm, small targets with gaps of below 1 cm might be susceptible to dose uncertainty caused by the MLC positioning error. For cases with OARs located close to or abutting with targets, the dose conformities of the MLC‐based plans were inferior to those of the cone/Iris‐based plans. This could be improved if the Multiplan utilizes intensity modulation for MLC‐based planning.

Although the CI values of the MLC‐based plans were slightly higher than those of the cone/Iris‐based plans, the beam delivery times of the MLC‐based plans were lower by approximately 30%–40%. Thus, the InCise1 MLC could be a useful modality for cases in which delivery time is a limiting factor or for multitarget cases. Van de Water et al.^(10)^ compared circular beams used in CyberKnife with arbitrary beam's eye view‐shaped fields to evaluate the time‐efficiency for dose delivery. The authors concluded that with a comparable conformity index, the delivery time of Iris plans was, on average, 40% longer than the delivery time generated by beam's eye view‐shaping devices such as a mini‐MLC for small targets (> 80 cm^3^) and up to 120% longer for larger targets. Although this study was designed for a CyberKnife with an arbitrary micro‐MLC, a time‐efficient alternative using a MLC system was suggested. For conventional linear accelerators, many studies reported that the advantage of VMAT was the efficiency of delivery rather than dosimetric improvement.^8^, ^24^, ^25^, ^26^ As shown in the present study, one of the significant advantages of the InCise1 MLC modality compared to the cone/Iris collimators was its superiority in the reduction of delivery time rather than the dosimetric advantages.

Similar to our study, McGuinness et al.^(13)^ evaluated the clinical advantages of the InCise1 MLC in the treatment of brain and prostate cancer patients. However, the authors concluded that the CI values of the MLC‐based plans were comparable to those of circular collimators, and delivery times were reduced almost by half. The McGuinness study showed that doses to the optical chiasm and brainstem were reduced for the MLC‐based plans. Although McGuinness and colleagues did not describe intracranial target locations relative to the OARs, the maximum dose to brainstem for five MLC‐based plans was in the range of 0.31 Gy to 5.75 Gy, showing that the targets were not abutted with the OARs since the dose to brainstem was a small fraction of the prescription dose of 10∼25Gy. On the other hand, we purposely selected intracranial cases with targets abutting with the OARs, as shown in Fig. 3. For targets abutting with OARs, the target coverages of the MLC‐based plans were inferior to those of the cone/Iris‐based plans with the same dose sparing of OARs.

Since the nominal dose rate of the CyberKnife M6 System is 1,000 MU/min, total treatment time is primarily dependent on the total number of beams rather than the total number of MUs; this suggests that there are opportunities to improve the delivery efficiency by finding ways to reduce the total number of beams without compromising the target coverage and dose conformity. Usually, robot travelling time between preset node positions takes longer than a reorientation of the machine between beams at a preset node.^(10)^ Therefore, the robot travel time could be reduced by minimizing the number of nodes. As shown in Table 4, the median number of segments in the MLC‐based plans was about 50% of the cone/Iris‐based plans, but the median number of beams of the MLC‐based plans was lower than the cone/Iris‐based plans by about 35%, resulting in a reduction in delivery time of about 35%.

For the InCise1 MLC, the maximum number of nodes is determined by the type of the path chosen. Head path, which consists of 73 or 171 nodes depending on the selection of short path or long path, is available to treat intracranial lesions. However, the total number of MLC segments generated by the treatment planning system is assigned by the optimization engine based on the number of nodes selected, the number of avoidance regions that intersect the target, and types of segment shapes chosen. As shown in Fig. 4, the plan quality did not vary significantly with increasing number of nodes in MLC‐based plans, but the delivery time increased considerably with increasing number of nodes. Additionally, the number of segments usually depended on the maximum MU per segment, and the delivery time increased as the maximum MU per segment decreased. The total number of segments per beam in the MLC‐based plans was, however, considerably less than the number of control points per arc in conventional VMAT delivery.

During the sequential optimization, we also tested MLC‐based plans with small MU per segment (50 MU/segment and 200 MU/node) to mimic IMRT in step‐and‐shoot delivery mode. By mimicking step‐and‐shoot delivery, the average number of segments per beam increased from 1∼2 segments to 4∼5 segments. As the MU per segment decreased, the estimated delivery time increased without any significant improvement in plan quality.

A reasonable starting point for optimization for generating an acceptable MLC‐based plan would be to start with the following parameters: two segments per node; maximum MU/segment ≈ 10% of maximum target dose; maximum MU/node ≈ 1.5 times the maximum MU/segment, and the maximum number of nodes selected ≈ 70∼100.

In terms of optimization, MultiPlan uses sequential optimization,^(15)^ which currently does not utilize intensity‐modulated optimization. Although the optimization is performed with the inverse planning technique with dose constraints to targets and OARs, it is, technically, similar to three‐dimensional conformal radiation therapy (3D CRT) with many noncoplanar beams or field‐in‐field dose painting, and therefore, the dose conformity in MLC‐based plans gets worse as target shapes become irregular. The inferior dose conformity of MLC plan partly results from the static delivery of the InCise1 MLC rather than sliding window or arc‐style delivery. However, the beam delivery time could be lowered significantly since total number of beams could be reduced using the InCise1 MLC.

Currently, the FSPB model is the only available dose engine for the MLC and the Monte Carlo dose calculation model is not available yet. Since the pencil beam model is known to overestimate dose in lung regions or close to air cavities,^27^, ^28^ the InCise1 MLC modality should be limited to cases where beams do not pass through low‐density areas, such as air cavities or lung.^(13)^

The InCise1 MLC leaves are single focused: their sides are aligned with beam divergence. The leaves are intentionally rotated by 0.5° to minimize the interleaf leakage rather than the tongue‐and‐groove design of other vendors. In the direction of leaf motion, the leaf is focused only when it is fully open, fully closed, or at midline, which means that any locations between fully open and midline are not fully focused. As the point of interest moves away from the midline, penumbra leaks into the neighboring field which may cause an increase in leakage radiation.

For the CyberKnife M6, there are no jaws to minimize leakage radiation through the MLC leaves. In conventional linear accelerators, secondary jaws with radiation transmission of 0.1% are usually set to the maximum field aperture of the target with extra margin and MLC leaves deliver radiation with intensity modulation within the field apertures set by jaws.^(29)^ For CyberKnife M6, there is no secondary jaw and, therefore, the InCise1 MLC might deliver additional leakage radiation to the surrounding normal tissue. However, the machine specification of the leakage radiation is set to below 0.5% compared to the center of the open field dose, and the total leakage radiation to the surrounding tissues will be clinically not significant.

Regarding treatment planning, MLC segments must conform to certain criteria to be modeled with the FSPB model. Only one continuous opening per segment is permitted, and every segment must have a minimum area of $56.25\;{\text{mm}}^{2}$ projected at 800 mm SAD. Additionally, the minimum width for an individual open leaf is 5 mm, and any opening along a line perpendicular to the direction of leaf motion must be at least three leaf widths wide to satisfy the required width of 7.5 mm. The limitations above contribute to the inferior quality of MLC‐based plans for irregularly shaped targets compared with cone/Iris‐based plans. For irregularly shaped targets, the InCise1 MLC could deliver with shorter delivery time, but the target coverage or dose conformity in the MLC‐based plans might be inferior to the cone or Iris‐based plans.

While Treuer et al.^(30)^ used simple and repeatable optimization scripts in all cases, optimization process in current study was not repeatable due to the use of dose volume lower limit (DVL) optimizer multiple times, as shown in Fig. 1. Additionally, the sequential minimal optimization, which uses random variables for optimization, should be considered as limitation in this plan comparison study.

As Dieterich and Pawlicki^(31)^ pointed out, many QA tools for the CyberKnife have to be developed on site. Patient‐specific QA procedures should be implemented to ensure the overall delivery accuracy as stated in the American Association of Physicists in Medicine (AAPM) Task Group Reports 101 and 135 recommendations.^15^, ^21^ During the QA delivery, six degree couch correction was not available for most cases since the imaging center of the QA plans was automatically set to that of clinical plans by the treatment planning system. For off‐centered targets, the X‐ray imaging system with 17cm±17cm field of view could not detect all of the fiducials in the Standard Imaging phantom, and therefore, provided only translational correction rather than six degree couch correction. For some cases with a very lateral target close to the skull, no fiducials were visible in either of the kV flat panel detectors, and consequently, the QA plans could not be delivered. QA delivery time was comparable to patient treatment time since the beam‐on time was relatively short due to the high dose rate of 1,000 MU/min, so that most of QA delivery time was taken by the robot movement from beam to beam. For a complicated plan with long delivery time, QA delivery could be a challenging task in a busy clinic.

## V. CONCLUSIONS

The present study showed that the delivery of intracranial SRS using the InCise1 MLC was dosimetrically feasible, and beam delivery time could be reduced by 30%∼40% compared to cone/Iris modality. However, small targets (size <7.6mm×7.5mm) might not be good candidates for MLC‐based planning. For cases with OARs located close to or abutting target volumes, the dose conformity of MLC‐based plan was inferior to that of cone/Iris‐based plan. Overall, the InCise1 MLC is a useful delivery modality for cases in which delivery time is a limiting factor or for multitarget cases.

## COPYRIGHT

This work is licensed under a Creative Commons Attribution 3.0 Unported License.
